# How the World Health Organization developed the *Guide for Action*

**Published:** 2023-01-30

**Authors:** Andreas S Mueller, Alarcos Cieza, Stuart Keel

**Affiliations:** Technical Advisor: Sensory Functions, Disability and Rehabilitation Unit, World Health Organization, Geneva, Switzerland.; Coordinator: Sensory Functions, Disability and Rehabilitation Unit, World Health Organization, Geneva, Switzerland.; Technical Officer: Sensory Functions, Disability and Rehabilitation Unit, World Health Organization, Geneva, Switzerland.

## Abstract

The World Health Organization is often called upon to develop global guidance; here is how a range of evidence and expertise was used to develop one such guide.

Member States of the World Health Organization (WHO) adopted a resolution on integrated people-centred eye care at the 73rd World Health Assembly in 2021. The resolution urged Member States to implement this new approach to eye care in their own health systems, and tasked WHO with developing a set of tools and guidelines to support this process. This led to the development of the *Guide for Action* (the *Guide*), which was published in May 2022.^1^

## How was the *Guide* developed?

### Establishment of expert groups

WHO established groups made up of experts in the fields of public health and methodology, as well as clinical experts from the field of eye care. A total of 360 experts were selected based on recommendations from professional associations and existing WHO networks, and to ensure balanced representation with respect to gender, geographical region, and income setting; their declarations of interest were also assessed. The groups provided technical input throughout the process of developing the *Guide* and its accompanying tools.

### Scoping and systematic reviews

The groups, in collaboration with methodologists and academics from relevant disciplines, carried out literature reviews to identify the best available evidence that could inform the development of each tool. The literature reviews were published in well-known academic journals, which means they were subjected to independent and rigorous peer review.

### Expert consensus

A stepwise process was then carried out among each expert group to achieve consensus on the technical elements of the tools. This included obtaining input from experts via online surveys, hosting virtual group consultations, and getting independent written feedback. Decision making was guided by two criteria:

1. What is the evidence for each tool?
2. Are they practical, and could they be realistically implemented within low- and middle-income countries?

### Peer review

Each tool underwent peer review to obtain feedback and recommendations for revisions. Peer reviewers included individuals from relevant WHO departments as well as eye care and public health experts.

**Figure 1:**
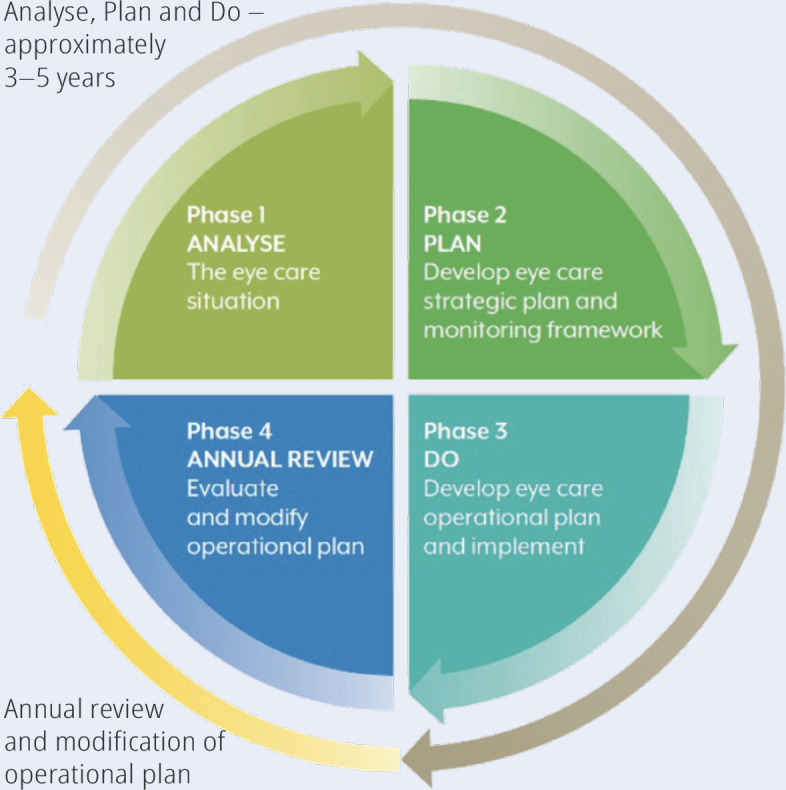
The WHO Guide for Action's Analyse, Plan, Do, Review cycle The *Guide for Action* offers step-by-step support to member countries to plan, implement, and monitor integrated people-centred eye care. It recommends that countries carry out an ‘Analyse–Plan–Do–Review’ cycle (Figure 1) by using the four tools developed alongside the *Guide*. The primary audiences for the *Guide* are governments of low- and middle-income countries (LMICs) and the agencies working with them; it is designed for use at the national level but can also be used at the sub-national level. For further information on the *Guide*, please visit: bit.ly/GuideAction. To learn more about integrated people-centred eye care, sign up for the course here: bit.ly/3EQQHMh

## Next steps

Government health planners and service providers, as well as non-governmental organisations supporting eye care, are now encouraged to use the *Guide* as needed.

In order to successfully implement action and improve eye care sustainably, it is important for governments to take lead in the implementation of the *Guide*, and for governments to ensure that any plans they develop are aligned and integrated within wider health plans and budgets.

## References

[1] Eye care in health systems: guide for action. Geneva: World Health Organization; 2022. bit.ly/GuideAction

